# Predicting postpartum haemorrhage: A systematic review of prognostic models

**DOI:** 10.1111/ajo.13599

**Published:** 2022-08-02

**Authors:** Bethany L. Carr, Maryam Jahangirifar, Ann E. Nicholson, Wentao Li, Ben W. Mol, Sharon Licqurish

**Affiliations:** ^1^ School of Nursing and Midwifery Monash University Melbourne Victoria Australia; ^2^ Faculty of Information Technology Monash University Melbourne Victoria Australia; ^3^ Department of Obstetrics and Gynaecology, The School of Clinical Sciences, Monash Health Monash University Melbourne Victoria Australia; ^4^ Monash Centre for Health Research & Implementation Monash Health Melbourne Victoria Australia

**Keywords:** postpartum haemorrhage, prognosis, pregnancy, risk factors, maternal mortality

## Abstract

**Background:**

Postpartum haemorrhage (PPH) remains a leading cause of maternal mortality and morbidity worldwide, and the rate is increasing. Using a reliable predictive model could identify those at risk, support management and treatment, and improve maternal outcomes.

**Aims:**

To systematically identify and appraise existing prognostic models for PPH and ascertain suitability for clinical use.

**Materials and Methods:**

MEDLINE, CINAHL, Embase, and the Cochrane Library were searched using combinations of terms and synonyms, including ‘postpartum haemorrhage’, ‘prognostic model’, and ‘risk factors’. Observational or experimental studies describing a prognostic model for risk of PPH, published in English, were included. The Critical Appraisal and Data Extraction for Systematic Reviews of Prediction Modelling Studies checklist informed data extraction and the Prediction Model Risk of Bias Assessment Tool guided analysis.

**Results:**

Sixteen studies met the inclusion criteria after screening 1612 records. All studies were hospital settings from eight different countries. Models were developed for women who experienced vaginal birth (*n* = 7), caesarean birth (*n* = 2), any type of birth (*n* = 2), hypertensive disorders (*n* = 1) and those with placental abnormalities (*n* = 4). All studies were at high risk of bias due to use of inappropriate analysis methods or omission of important statistical considerations or suboptimal validation.

**Conclusions:**

No existing prognostic models for PPH are ready for clinical application. Future research is needed to externally validate existing models and potentially develop a new model that is reliable and applicable to clinical practice.

## INTRODUCTION

Primary postpartum haemorrhage (PPH), defined as a loss of ≥500 mL of blood from the genital tract within 24 h of birth,^1^ remains one of the leading causes of maternal mortality^2^ and morbidity worldwide.^3^ Australia^4^, ^5^ and other high‐resource countries^6^, ^7^, ^8^ report a rising incidence in recent decades, along with an increase in severity^8^, ^9^, ^10^ and the morbidities associated with major blood loss.^4^, ^11^, ^12^ The impact of PPH on women and their families is also significant.^12^, ^13^

According to current guidelines, the management of PPH requires clinicians recognise risk indicators and promptly respond and treat excessive bleeding.^14^, ^15^, ^16^, ^17^ Diagnosis is challenging: blood loss is frequently underestimated,^18^ and individual risk factors that are identified in the literature are not always present.^14^, ^15^, ^18^

For healthcare systems, a risk‐stratified approach can better allocate resources and assist with recommending place of birth based on level of risk. Identifying absolute risk of the outcome requires the use of available data in a prognostic model. Prognostic models are frequently reported as being overly complex for everyday use.^19^ As electronic medical records become increasingly common, prognostic models could be readily applied in clinical practice^20^ by automatically extracting data from patient records and calculating risk in real time. Active prognostication could enable early intervention and reduce the severity of PPH.

Prognostic models are abundant in medicine, but their translation into clinical practice is not common.^19^ A systematic review of over 250 prediction models in obstetrics found the vast majority are not used in clinical practice and few report on their performance or impact on patient outcomes.^21^ A recent systematic review by Neary *et al*.^22^ of predicting risk of PPH as well as the risk of blood transfusion, identified 14 prognostic models, although none were considered ready for clinical use due to high risk of bias, lack of validation (internal and external) and limitations of target populations not being applicable to the general obstetric population.

This systematic review was conducted to establish the existing literature of prognostic models for PPH and inform progress toward optimal primary research and translation into clinical practice. The aims of this systematic review were to: identify models for prediction of PPH; describe the characteristics of the models; compare their performance; and critically assess the conduct and reporting of the prediction modelling development methods.

## MATERIALS AND METHODS

This review was conducted and reported in accordance with the Preferred Reporting Items for Systematic Reviews and Meta‐analysis (PRISMA) guidelines^23^ and was registered with the International Prospective Register of Systematic Reviews (PROSPERO), number CRD42020136926.^24^ Table S1 shows the inclusion and exclusion criteria for potentially eligible studies; a protocol was not prepared. Keywords and synonyms were developed (see Table S3), and combinations of the relevant Medical Subject Heading (MeSH) terms, with truncations, keywords and word variants for ‘postpartum haemorrhage’, ‘prognostic model’, and ‘risk factors’.

MEDLINE, CINAHL, Embase and the Cochrane Library databases were searched from inception to May 2019, with an update on 16 March 2020. Reference lists of relevant articles were searched manually to identify additional papers. Identified articles were uploaded into Covidence systematic review software (Veritas Health Innovation, Melbourne, Australia, available at http://www.covidence.org) and screened independently in duplicate by two or more reviewers against the inclusion and exclusion criteria, and conflicts were resolved by a third reviewer. Ethics approval was not applicable as this is a systematic review.

We extracted data using the Critical Appraisal and Data Extraction for Systematic Reviews of Prediction Modelling Studies (CHARMS).^25^ Data extracted included: source of data; participants; outcome to be predicted; candidate predictors; sample size; missing data; model development; model performance; model evaluation; results and interpretation. We sought to extract the predictive performance of each model by using whatever statistical measures they reported. These measures included any summaries of discrimination and calibration. Discrimination was defined as the extent to which predicted risks discriminate between participants with or without the outcome (PPH), and calibration defined as the extent to which predicted risks correspond to observed risks.^26^ Data were extracted independently by two reviewers and conflicts checked by a third.

Results of the included models are presented with whatever measures were used to report performance of the model. Core outcome sets for evaluating interventions for PPH have been published by Meher and colleagues,^27^ and although we have not evaluated interventions, we have taken their recommendations under consideration.

We completed risk of bias assessment according to the Prediction Model Risk of Bias Assessment Tool (PROBAST).^26^ Domains that relate to the key methodological concerns of prognostic research were assessed. Any discrepancies in risk of bias were discussed between the reviewers, and any remaining conflicts were resolved by a third reviewer.

## RESULTS

### Study selection

**Figure 1:**
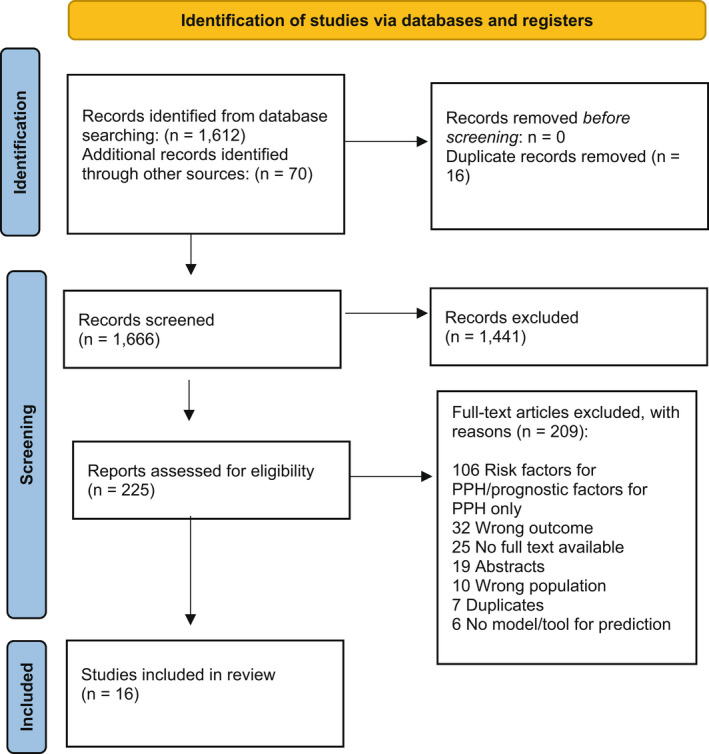
Preferred Reporting Items for Systematic Reviews and Meta‐analysis (PRISMA) flowchart of included studies. PPH, postpartum haemorrhage.

Our search retrieved 1612 records. Following removal of duplicates and screening of titles and abstracts, 225 full text articles were assessed for eligibility (Fig. 1). Initially 19 studies reporting a prognostic model for PPH were included. After further analysis, three were excluded due to having a prognostic model predicting risk of severity of PPH^28^, ^29^ and need for advanced interventional procedures^30^ once PPH had already been diagnosed. Therefore, 16 studies were included in this review.

### Study characteristics

Of the 16 included studies, nine developed more than one model. These studies evaluated different sets of candidate predictors such as clinical and radiomic features,^31^ to separate antenatal predictors and intrapartum,^32^ or different cut points for haemoglobin (Hb) levels.^33^ In this review, we present the models collectively according to each study. All the studies included in this review reported the development of prediction models (Table S2); of the 16, only two present development models with external validation in independent data.^31^, ^33^ No studies validating previously developed models were identified.

### Source of data and participants

Thirteen of the included studies had a retrospective cohort design (Table S2) and three were prospective design. One study used trial data,^32^ two performed a secondary analysis on data from prospective studies.^34^, ^35^ One study was a retrospective case–control design^36^ and another retrospectively analysed data from a previous case–control study.^37^

The study populations varied across studies (Table 1). Three studies included any mode of birth,^32^, ^36^, ^38^ one of these limited their population to women with gestational hypertension or mild pre‐eclampsia.^32^ Seven studies included women with vaginal birth only^33^, ^34^, ^35^, ^37^, ^39^, ^40^, ^41^ the remaining studies included women with caesarean birth^31^, ^42^, ^43^, ^44^, ^45^, ^46^ three of which limited their populations to those with abnormalities of the placenta (described as low lying,^46^ placenta accrete spectrum disorder,^31^ and placenta praevia^45^). The number of participants in the studies ranged from 40 to 53 438.

**Table 1:** Clinical characteristics of study populations including outcome measurement and sample size

Study	Model	Mode of birth	Outcome to be predicted	Inclusion criteria	Sample size: No. for development set, (No. with outcome)	Sample size: No. for validation set, (No. with outcome)
Chen *et al*. (2011)	Predict risk of PPH based on prior hospital admissions for chronic diseases	Any	Obstetric haemorrhage diagnosis and procedure codes as classified by the 10th revision of International Classification of Diseases ‐ Australian Modification (ICD‐10‐AM)	All women having their first baby during study period	*n* = 53 438 (5047)	Not applicable
Helman *et al*. (2015)	Predict risk of major PPH	Any	Major obstetric haemorrhage defined as transfusion of ≥5 units PRBC within 48 h following birth	All women with major obstetric haemorrhage and no documented coagulation disorders, matched to controls	*n* = 122 cases *n* = 488 controls	Not applicable
Koopmans *et al*. (2014)	Predict risk of PPH in women with gestational hypertension or mild pre‐eclampsia	Any	Blood loss >1000 mL within 24 h following delivery	Singleton, cephalic presentation, gestation 36–41 weeks, PIH or mild PE	*n* = 1132 (118)	*n* = 52 (52)
Prata *et al*. (2011)	Predict risk of PPH using available antenatal and intrapartum variables	Vaginal	Blood loss ≥500 mL measured every 20 min for the first 4 h following birth	Singleton, anticipated vaginal birth, gestation >36 weeks	*n* = 2510 (93)	Not applicable
Biguzzi *et al*. (2012)	A nomogram to predict PPH for women having NVB at term gestation	Vaginal	Blood loss ≥500 mL after birth	Women with singleton pregnancy, term gestation (≥37 weeks), NVB	*n* = 6011 (1435)	Not applicable
Peyvandi *et al*. (2012)	Predict risk of PPH using prepartum fibrinogen levels	Vaginal	Blood loss ≥500 mL after birth	Women with singleton pregnancy, term gestation (≥37 weeks), NVB, prepartum fibrinogen levels	*n* = 4461 (1076)	Not applicable
Niepraschk‐von Dollen *et al*. (2016)	Predict severity of PPH based on fibrinogen levels prior to birth	Vaginal	Blood loss ≥500 mL, severe PPH ≥1000 mL	>18 years, live, singleton pregnancy at 37+ weeks, vaginal birth	*n* = 689 (106)	Not applicable
Rubio‐Alvarez *et al*. (2018)	Predict risk of excessive bleeding	Vaginal	Excessive blood loss measured as a reduction in Hb >3.5 g/dL from the start of birth to 24 h post	Singleton pregnancy, gestation >35 weeks, NVB, liveborn infant	*n* = 2336 (197)	*n* = 953 (63)
Tsu (1994)	Predict risk of PPH and CPD	Vaginal	Blood loss ≥600 mL after birth	Singleton, vertex presentation, spontaneous onset of labour	Controls *n* = 299 CPD *n* = 203 PPH *n* = 151	Not applicable
Sittiparn & Siwadune (2017)	Risk score for PPH based on medical history and clinical characteristics	Vaginal	Blood loss ≥500 mL after birth	Singleton, vertex presentation, NVB	*n* = 650 (325)	Not applicable
Suta *et al*. (2015)	Risk score for PPH at CS	Caesarean	EBL ≥1000 mL at CS	Singleton pregnancy at any gestation with CS	*n* = 2405 (244)	Not applicable
Dunkerton *et al*. (2017)	Predict risk of developing PPH during CS	Caesarean	Blood loss ≥1000 mL	CS birth	*n* = 18 172 (2997)	*n* = 6058 (NR)
Lee *et al*. (2018)	Scoring model to predict massive PPH for women with placenta praevia	Caesarean	Massive PPH defined as one of the following: blood loss >2000 mL during surgery; postpartum transfusion of 4 or more pints of PRBC; caesarean hysterectomy; or uterine embolisation triggered by postpartum bleeding	Singleton, >24 weeks gestation with placenta praevia	*n* = 506 (73)	Not applicable
Sei *et al*. (2018)	Size of leiomyoma to predict massive PPH during CS	Caesarean	Blood loss ≥1000 mL after CS	Women undergoing CS with no labour prior to birth, no placental abnormalities or haemorrhagic diseases	*n* = 759 (182)	Not applicable
Shinohara *et al*. (2018)	Neonatal birthweight to predict massive PPH in women with LLP	Caesarean	EBL >1500 mL during and up to 2 h following CS	Women with LLP	*n* = 40 (15)	Not applicable
Wu *et al*. (2019)	A nomogram using radiomic features and medical history to predict PPH in women undergoing CS with PAS	Caesarean	EBL >1000 mL during CS	Singleton pregnancies suspected with PAS disorders who underwent MRI for placenta evaluation, caesarean birth, and had recorded EBL and transfusion protocol	*n* = 207 (102)	*n* = 91 (41)

Abbreviations: CPD, cephalopelvic disorder; CS, caesarean section; DM, diabetes mellitus; EBL, estimated blood loss; Hb, haemoglobin; IOL, induction of labour; LLP, low lying placenta; MRI, magnetic resonance imaging; NR, not reported; NVB, normal vaginal birth; PAS, placenta accrete spectrum; PE, pre‐eclampsia; PIH, pregnancy induced hypertension; PPH, postpartum haemorrhage; PRBC, packed red blood cells.

### Outcome to be predicted

Although the outcome was primary PPH, there was variation in the measurement or description of this outcome among the studies (Table 1). One study^38^ relied on medical record coding of obstetric haemorrhage using the 10th revision of International Classification of Diseases ‐ Australian Modification (ICD‐10‐AM). Four studies^32^, ^34^, ^40^, ^41^ measured blood loss using a calibrated drape or plastic basin, yet their definitions of PPH include ≥500 mL in the first 24 h after birth, ≥500 mL in the first four hours after birth, and severe PPH as ≥1000 mL. Two studies^35^, ^39^ described PPH as an estimated blood loss (EBL) of ≥500 mL, and one^37^ as ≥600 mL after vaginal birth without describing how it was measured. Most of the studies that included women having a caesarean birth defined PPH as a blood loss ≥1000 mL,^31^, ^42^, ^43^, ^44^ and one defined it as ≥1500 mL within two hours from birth.^46^ One study defined PPH as excessive postpartum blood loss defined as a reduction in Hb >3.5 g/dL up to 24 h post‐birth.^33^ One study that was predicting risk of massive PPH for women with placenta praevia defined their outcome as any one of the following: EBL ≥2000 mL; transfusion of ≥4 packed red blood cells (PRBC); caesarean hysterectomy; or uterine arterial embolisation triggered by postpartum bleeding.^45^ Another study predicting risk of major obstetric haemorrhage did not report a measured blood loss, rather they assessed their outcome as women who had a transfusion of ≥5 units PRBC during hospitalisation for the birth.^36^

### Candidate predictors

The number of candidate predictors ranged 1–20 (Table 2). The types of selected variables included maternal demographics (such as maternal age,^31^, ^32^, ^33^, ^34^, ^37^, ^39^, ^41^, ^43^, ^44^, ^45^ body mass index^32^, ^33^, ^39^, ^42^, ^43^ and maternal weight,^41^ race^44^ and ethnicity^32^, ^41^ [one study specified only Asian ethnicity^42^]); medical and obstetric history (such as parity,^32^, ^33^, ^34^, ^36^, ^37^, ^40^, ^41^, ^42^, ^43^, ^44^ history of caesarean section,^31^, ^36^, ^42^ diabetes^38^, ^39^ hypertensive disorders [pre‐existing^38^ or developed during pregnancy^32^, ^39^, ^42^]); results from investigations (laboratory,^31^, ^32^, ^33^, ^35^, ^37^, ^40^, ^41^ ultrasound,^31^, ^43^, ^45^, ^46^ or magnetic resonance imaging [MRI] results^31^); and obstetric variables relating to the pregnancy, labour and mode of birth.^32^, ^33^, ^34^, ^36^, ^40^, ^41^, ^42^, ^44^ Predictors relating to the third stage of labour included: active management of the third stage of labour^33^, ^34^ time after atony;^40^ retained placenta;^41^ manual removal of the placenta;^33^ and third stage abnormality.^36^ Seven studies included predictors that are known after the birth, such as neonatal birthweight.^33^, ^34^, ^40^, ^41^, ^43^, ^44^, ^46^

**Table 2:** Selected predictors, evaluation and performance of the models; overall risk of bias rating

Study	Model	Selected candidate predictors	Evaluation	Calibration	Performance discrimination, *c*‐statistic (95% CI)	Overall risk of bias
Chen *et al*. (2011)	Predict risk of PPH based on prior hospital admissions for chronic diseases	ICD‐10‐AM diagnosis and procedure codes for 8 chronic diseases: cardiac disease, chronic kidney disease, asthma/chronic obstructive pulmonary disease, psychiatric disorders, pre‐existing hypertension, pre‐existing diabetes, thyroid disorders, and autoimmune disease	Apparent performance only	NR	0.624	High
Helman *et al*. (2015)	Predict risk of major PPH	Grand multiparity, previous CS, ≥3 miscarriages, multiple pregnancy, IOL, instrumental delivery, CS, 3rd stage abnormality	Apparent performance only	GOF (*P* = 0.646)	0.919 (0.890–0.48)	High
Koopmans *et al*. (2014)	Predict risk of PPH in women with gestational hypertension or mild preeclampsia	Maternal age, parity, smoking, BMI, ethnicity, education level, previous abortion, BP and laboratory results, diagnosis or gestational hypertension or PE, gestational age, pain relief, duration of 1st and 2nd stages of labour, use of prostaglandins, oxytocin, magnesium sulfate, onset of labour, mode of delivery, perineal rupture or episiotomy	Internal validation by resampling (bootstrap) and repeat analysis with need for blood transfusion	Development: GOF (*P* = 0.26 antepartum); GOF (*P* = 0.36 intrapartum) Validation: GOF (*P* = 0.82 antepartum); GOF (*P* = 0.54 intrapartum)	Development: 0.59 (0.53–0.64, antepartum) 0.64 (0.59–0.70, intrapartum) Validation: 0.69 (0.62–0.77, antepartum) 0.75 (0.68–0.81, intrapartum)	High
Prata *et al*. (2011)	Predict risk of PPH using available antenatal and intrapartum variables	Maternal age >30, literacy, parity, ANC, Hx of PPH, Hx obstetric complications, intact membranes, anaemia, cervical dilation on admission, type of birth, episiotomy, augmentation, complete placental expulsion, vaginal tears, neonatal birthweight, length of stages of labour, AMTSL	Apparent performance only	NR	NR	High
Biguzzi *et al*. (2012)	A nomogram to predict PPH for women having NVB at term gestation	Maternal age, maternal weight, parity, Hb, platelets, neonatal birthweight, placental weight, vacuum extraction, Kristellar manoeuvre, lacerations, episiotomy, retained placental, and ethnicity	Internal validation by resampling (bootstrap) 200 replicates	Calibration plot demonstrated overall good performance	0.70 (after correcting for optimism)	High
Peyvandi *et al*. (2012)	Predict risk of PPH using prepartum fibrinogen levels	Fibrinogen level	Apparent performance only	NR	0.51 (0.49–0.53)	High
Niepraschk‐von Dollen *et al*. (2016)	Predict severity of PPH based on fibrinogen levels prior to birth	Fibrinogen, parity, birthweight >4000 g, genital tract laceration, time after atony, instrumental delivery, episiotomy	Apparent performance only	NR	0.502 (0.433–0.570, ≥500 mL EBL) 0.665 (0.548–0.782, ≥1000 mL EBL)	High
Rubio‐Alvarez *et al*. (2018)	Predict risk of excessive bleeding	Instrumental birth, AMTSL, manual removal of placenta, episiotomy, primiparity, neonatal birth weight, maternal age, length of first and second stages of labour, antepartum Hb	External (same centre in a different time period)	NR	Development: 0.90 (0.86–0.94, final model, Hb <8 g/dL) Validation: 0.84 (0.74–0.93 final model Hb <8 g/dL)	High
Tsu (1994)	Predict risk of PPH and CPD	Maternal age >35, low parity (0, 1), poor obstetric outcome last pregnancy, antenatal Hb <12 g/dL, antenatal hospitalisation for obstetric condition	Apparent performance only	NR	NR	High
Sittiparn & Siwadune (2017)	Risk score for PPH based on medical history and clinical characteristics	Maternal age ≥35, pre‐pregnancy BMI ≥25 kg/m^2^, PIH, Type 2 DM	Apparent performance only	NR	0.660 (0.54–0.78)	High
Suta *et al*. (2015)	Risk score for PPH at CS	Maternal age ≥35, race (Asian other than Thai), multiparity, placenta praevia, neonatal birthweight ≥4000 g, emergency CS, abnormal 2nd stage of labour, cervical dilatation at time of birth	Apparent performance only	NR	0.647 (0.61–0.68)	High
Dunkerton *et al*. (2017)	Predict risk of developing PPH during CS	Placenta praevia, previous CS, APH, BMI ≥35, emergency CS, Asian ethnicity, multiple pregnancy, grand multiparity, PIH/PE	Training test split	NR	NR	High
Lee *et al*. (2018)	Scoring model to predict massive PPH for women with placenta praevia	Maternal age ≥35, antepartum bleeding, non‐cephalic presentation, complete placenta praevia, anterior placenta, multiple lacunae, uteroplacental hypervascularity	Apparent performance only	NR	0.856	High
Sei *et al*. (2018)	Size of leiomyoma to predict massive PPH during CS	Maternal age >35, BMI ≥25 kg/m^2^, gestation ≥38 weeks, primipara, neonatal birth weight ≥2500 g, volume of largest leiomyoma ≥175 cm^3^, number of leiomyomas ≥3	Internal validation by resampling (bootstrap) of 1000 replicates	NR	NR	High
Shinohara *et al*. (2018)	Neonatal birthweight to predict massive PPH in women with LLP	Neonatal birthweight	Apparent performance only	NR	0.74	High
Wu *et al*. (2019)	A nomogram using radiomic features and medical history to predict PPH in women undergoing CS with PAS	Maternal age, number of previous CS, Hb value prior to CS, radiomic signature	External, one other centre in China, 3‐fold cross validation	Development: GOF (*P* = 0.181) Validation: GOF (*P* = 0.165)	Development: 0.888 (0.844–0.933, clinic‐radiomic model) Validation: 0.832 (0.746–0.913, clinic‐radiomic model)	High

Abbreviations: AMTSL, active management of the third stage of labour; ANC, antenatal care; APH, antepartum haemorrhage; BMI, body mass index; BP, blood pressure; CPD, cephalopelvic disorder; CS, caesarean section; DM, diabetes mellitus; EBL, estimated blood loss; GOF, goodness of fit; Hb, haemoglobin; Hx, history; ICD‐10‐AM, International Classification of Diseases ‐ Australian Modification; IOL, induction of labour; LLP, low lying placenta; NVB, normal vaginal birth; NR, not reported; PAS, placenta accrete spectrum; PE, pre‐eclampsia; PIH, pregnancy induced hypertension; PPH, postpartum haemorrhage.

### Model development

Multivariable logistic regression was used in all studies except for one which used a non‐parametric recursive partitioning algorithm.^42^ In this study the authors developed a binary decision‐tree that uses answers from a series of yes/no questions about clinical characteristics to predict risk of PPH at caesarean.

The presence and handling of missing data was frequently omitted from analysis, only Koopmans *et al*.^32^ reported using multiple imputation methods to deal with missing data.

### Predictors selected in final models

Predictors selected for the models were frequently the same across the studies. In ten of the 16 studies,^31^, ^32^, ^33^, ^34^, ^37^, ^39^, ^41^, ^43^, ^44^, ^45^ maternal age was selected in the model. Parity was also frequently selected.^32^, ^33^, ^34^, ^36^, ^37^, ^40^, ^41^, ^42^, ^43^, ^44^ The most frequently included test results were blood tests, measuring fibrinogen levels,^35^, ^40^ platelets^32^, ^41^ and haemoglobin.^31^, ^32^, ^33^, ^37^, ^41^

### Model evaluation and predictive performance

Model performance was evaluated using the same dataset (apparent performance only) for most of the included studies (Table 2). Three studies^32^, ^41^, ^43^ reported using internal validation with bootstrap resampling methods. Koopmans *et al*.^32^ also validated their findings by repeating the analysis with the need for blood transfusion. Two studies^31^, ^33^ evaluated their models with external validation; Rubio‐Alvarez *et al*.^33^ used data from the same centre from a different period of time (temporal validation), and Wu *et al*.^31^ used data from two centres in Zhengzhou, China to develop and validate their models (geographical validation). One study^42^ used a ‘training test split’ by randomly dividing the data into development and validation samples. Model performance was most commonly reported in terms of discrimination with 12 of the studies reporting a concordance statistic (*c*‐statistic) for their final models. For those reporting it, the range was 0.502–0.919 (development cohorts), and 0.69–0.84 (validation cohorts). Three studies^31^, ^32^, ^36^ provided an assessment of calibration with non‐significant findings for the Hosmer‐Lemeshow goodness of fit test.

Nine studies^31^, ^33^, ^34^, ^37^, ^39^, ^40^, ^44^, ^45^, ^46^ reported classification measures of sensitivity and specificity and/or positive and negative predictive values.

As assessed using the PROBAST^26^ tool, all studies were rated high risk of bias overall (Table 3). All studies rated a high risk of bias for the analysis domain. This was due to use of inappropriate analysis methods or omission of important statistical considerations. The performance of prediction models is to some extent overestimated when model development and performance assessment use the same data set; this was the case for all of the studies except for the two^31^, ^33^ which used an external dataset for validation. Risk of bias is also indicated when predictive performance is not reported with both discrimination and calibration. Twelve of the included studies did not report measures of calibration. Ten studies did not account for model overfitting and optimism with internal validation. The handling of missing data was omitted in the majority of studies; only one study reported using multiple imputation for missing variables in their methodology.^32^ In the participant domain of the PROBAST^26^ tool Tsu^37^ and Helman *et al*.^36^ were both rated high risk of bias for using non‐nested case–control design. Lee *et al*.^45^ and Sei *et al*.^43^ rated high risk of bias for the predictor domain, as both relied on predictors that required subjective interpretation by different assessors. Four studies^34^, ^41^, ^44^, ^46^ rated high risk of bias for using predictors that may not be available at the time the model is intended to be used (eg neonatal birthweight and placental weight). Peyvandi *et al*.^35^ relied on a predictor that was not assessed in the same way for all participants, therefore was also rated high risk of bias. Tsu^37^ was classified as unclear as we were unable to determine if predictor assessments were made without knowledge of the outcome data or if all predictors were available at the time the model was intended to be used.

**Table 3:** Risk of bias

Study	Risk of bias				Applicability			Overall	
	Participants	Predictors	Outcome	Analysis	Participants	Predictors	Outcome	Risk of bias	Applicability
Chen *et al*. (2011)	+	+	+	−	+	−	−	−	−
Helman *et al*. (2015)	−	+	+	−	+	+	+	−	+
Koopmans *et al*. (2014)	+	+	+	−	+	+	+	−	+
Prata *et al*. (2011)	+	−	+	−	+	−	+	−	−
Biguzzi *et al*. (2012)	+	−	+	−	+	+	+	−	+
Peyvandi *et al*. (2012)	+	−	+	−	+	+	+	−	+
Niepraschk‐von Dollen *et al*. (2016)	+	+	+	−	+	+	+	−	+
Rubio‐Alvarez *et al*. (2018)	+	+	+	−	+	+	+	−	+
Tsu (1994)	−	?	−	−	+	?	+	−	?
Sittiparn & Siwadune (2017)	+	+	+	−	+	+	+	−	+
Suta *et al*. (2015)	+	−	−	−	+	−	+	−	−
Dunkerton *et al*. (2017)	+	+	−	−	+	+	+	−	+
Lee *et al*. (2018)	+	−	−	−	−	+	+	−	−
Sei *et al*. (2018)	+	−	+	−	+	−	+	−	−
Shinohara *et al*. (2018)	+	−	−	−	−	−	+	−	−
Wu *et al*. (2019)	+	+	−	−	−	?	+	−	−

(+) Indicates low risk of bias; (−) indicates high risk of bias; (?) indicates unclear risk of bias.

## DISCUSSION

We found 16 papers reporting the development of prognostic studies for PPH. Evaluation of performance and clinical impact is limited, as most of the models were not externally validated. All of the models had high risk of bias, in terms of validity and applicability, according to the PROBAST^26^ criteria. Highlighted concerns include the selection of predictors, mostly in relation to how they were selected (such as from univariate analysis) and their poor applicability as some are not routinely available data (such as MRI results) and the inclusion of variables that are known after PPH has most likely occurred (such as neonatal birthweight).

For a prognostic model to be usable in the clinical setting, all the included predictors need to be available at the time the model is intended to be used (the time of prediction)^26^ and the model needs to undergo wide external validation because models without appropriate validation tend to be biased by overestimating the relative performance.^25^ Most studies identified in this review did not specify at what time point the model should be used, although Koopmans *et al*.^32^ developed two models, one using antenatal variables, and one combining antenatal with intrapartum variables. They evaluated the performance of their model with resampling techniques (bootstrap) and validated this with the need for blood transfusion. Prata *et al*.^34^ also developed a version of antenatal and intrapartum models but combined these with the components of active management of the third stage of labour (AMTSL) to determine which has the greatest impact on PPH. This paper, like many of the included studies in this review, did not validate the performance of the model.

Some of the studies included in this review were not explicitly reported as prognostic model studies. Development of a multivariable prognostic model involves the following steps:^47^ identifying the important predictors, assigning relative weights to each predictor, estimating the model's predictive performance through calibration and discrimination and its potential for optimism using internal validation techniques, and adjusting for overfitting. Chen *et al*.^38^ aimed to examine the effects of increased ascertainment on modelling of risk factors for obstetric haemorrhage by looking at chronic disease history in medical records; they did not assign weights to predictors or evaluate their performance; nor did Peyvandi *et al*.^35^ and Niepraschk‐von Dollen *et al*.^40^ who both evaluated the relationship between PPH and antenatal fibrinogen levels. Helman *et al*.^36^ evaluated modifiable and non‐modifiable risk factors for major obstetric haemorrhage; they calibrated their model using the Hosmer‐Lemeshow test for logistic regression, and they used a case–control design, neither of which are recommended for the development of a prognostic model.^25^ The above‐mentioned studies did not explicitly aim to build a predictive model for clinical use, although they followed some but not all of the required steps. In addition to Prata *et al*.,^34^ another six studies^37^, ^39^, ^42^, ^44^, ^45^, ^46^ aimed to develop a model to predict PPH but did not validate the performance of the model.

The strengths of this review include prospectively registering in PROSPERO prior to conducting the search of the literature. Secondly, the search strategy was thorough and used reliable tools such as CHARMS^25^ and PROBAST^26^ to guide a critically evaluative approach. The analysis of the data extracted, and assessment of risk of bias and applicability were systematic. We relied on a universally accepted definition of primary PPH^1^ and only included models predicting excessive postpartum blood loss. We did not include studies predicting risk of blood transfusion as this may or may not be as a result of PPH. This ensured we did not include models predicting the effectiveness of interventions and treatment for PPH. Furthermore, this review was able to identify models that were not identified in a previous published review.^22^

The findings of this review are limited by the quality of the included studies. Overfitting and optimistic estimates of performance is a concern with most of the studies included in this review as ten of the studies quantified the predictive ability of their models in the same data used for the development of the model. Although all the included studies had the same outcome to be predicted, nuances in the definitions and timing of measurement meant that PPH included the following variances: visual estimation of blood loss, measured by volume in a calibrated drape, or a reduction in haemoglobin levels following blood loss. Timing of the measurement included two hours after the birth, four hours, or within 24 h after the birth. This is a source of heterogeneity across the studies. Variance in the target population has made applicability challenging; specific populations such as those with placental abnormalities or hypertensive disorders do not represent the general obstetric population.

This review was unable to identify any prognostic models that are ready for clinical application. We recommend future research in two steps: firstly, prognostic models must be developed and reported according to the Transparent Reporting of a multivariable prediction model for Individual Prognosis or Diagnosis (TRIPOD)^48^ guidelines to improve transparency and clinical usefulness of the model. Secondly, the models must be externally validated in different clinical settings to determine transportability and feasibility. If prognostic models are developed following these steps, then this may translate to early detection and appropriate management of PPH and improve outcomes for women.

## Funding

This study received no external or specific source funding.

## Supporting information


**Table S1.** Inclusion and exclusion criteria.

**Table S2.** Source of data and characteristic of studies used to develop models for predicting postpartum haemorrhage.

**Table S3.** Search terms.

## References

[1] Van Der Nelson H , O'Brien S , Lenguerrand E *et al*. Intramuscular oxytocin versus oxytocin/ergometrine versus carbetocin for prevention of primary postpartum haemorrhage after vaginal birth: Study protocol for a randomised controlled trial (the IMox study). Trials 2019; 20(1): 1–10. 30606246 10.1186/s13063-018-3109-2 PMC6319006

[2] Say L , Chou D , Gemmill A *et al*. Global causes of maternal death: A WHO systematic analysis. Lancet Glob Health 2014; 2(6): 323–333. 10.1016/S2214-109X(14)70227-X 25103301

[3] Al‐Zirqi I , Vangen S , Forsen L , Stray‐Pedersen B . Prevalence and risk factors of severe obstetric haemorrhage. BJOG An Int J Obstet Gynaecol 2008; 115(10): 1265–1272. 10.1111/j.1471-0528.2008.01859.x 18715412

[4] Ford JB , Patterson JA , Seeho SKM , Roberts CL . Trends and outcomes of postpartum haemorrhage, 2003‐2011. BMC Pregnancy Childbirth 2015; 15(1): 1–10. 10.1186/s12884-015-0788-5. 26670767 PMC4681164

[5] Cameron C , Roberts CL , Olive EC *et al*. Trends in postpartum haemorrhage. Aust N Z J Public Health 2006; 30(2): 151–156. 16681337 10.1111/j.1467-842x.2006.tb00109.x

[6] van Stralen G , von Schmidt auf Altenstadt JF , KWM B *et al*. Increasing incidence of postpartum hemorrhage: The Dutch piece of the puzzle. Acta Obstet Gynecol Scand 2016; 95(10): 1104–1110. 27460955 10.1111/aogs.12950

[7] Joseph KS , Rouleau J , Kramer MS *et al*. Investigation of an increase in postpartum haemorrhage in Canada. BJOG An Int J Obstet Gynaecol 2007; 114(6): 751–759. 10.1111/j.1471-0528.2007.01316.x 17516968

[8] Lutomski JE , Byrne BM , Devane D , Greene RA . Increasing trends in atonic postpartum haemorrhage in Ireland: An 11‐year population‐based cohort study. BJOG An Int J Obstet Gynaecol 2012; 119(3): 306–314. 10.1111/j.1471-0528.2011.03198.x 22168794

[9] Rossen J , Økland I , Nilsen OB , Eggebø TM . Is there an increase of postpartum hemorrhage, and is severe hemorrhage associated with more frequent use of obstetric interventions? Acta Obstet Gynecol Scand 2010; 89(10): 1248–1255. 20809871 10.3109/00016349.2010.514324

[10] Kramer MS , Berg C , Abenhaim H *et al*. Incidence, risk factors, and temporal trends in severe postpartum hemorrhage. Am J Obstet Gynecol [Internet] 2013; 209(5): 449.e1–449.e7. Available from:. 10.1016/j.ajog.2013.07.007. 23871950

[11] Merriam AA , Wright JD , Siddiq Z *et al*. Risk for postpartum hemorrhage, transfusion, and hemorrhage‐related morbidity at low, moderate, and high volume hospitals. J Matern Neonatal Med 2018; 31(8): 1025–1034. 10.1080/14767058.2017.1306050. PMC6112239 28367647

[12] Mehrabadi A , Hutcheon JA , Lee L *et al*. Trends in postpartum hemorrhage from 2000 to 2009: A population‐based study. BMC Pregnancy Childbirth 2012; 12(108): 1–9. 23057683 10.1186/1471-2393-12-108 PMC3534600

[13] Green L , Knight M , Seeney FM *et al*. The epidemiology and outcomes of women with postpartum haemorrhage requiring massive transfusion with eight or more units of red cells: A national cross‐sectional study. BJOG An Int J Obstet Gynaecol 2016; 123(13): 2164–2170. 10.1111/1471-0528.13831 26694742

[14] Safer Care Victoria , Postpartum haemorrhage‐ prevention, assessment and management [Internet]. 2019. [cited 2021 Feb 22]. Available from: https://www.bettersafercare.vic.gov.au/clinical‐guidance/maternity/postpartum‐haemorrhage‐pph‐prevention‐assessment‐and‐management#goto‐risk‐factors

[15] RANZCOG . Management of Postpartum Haemorrhage (PPH) [Internet]. 2017. Available from: https://ranzcog.edu.au/RANZCOG_SITE/media/RANZCOG‐MEDIA/Women%27sHealth/Statement%20and%20guidelines/Clinical‐Obstetrics/Management‐of‐Postpartum‐Haemorrhage‐(C‐Obs‐43)‐Review‐July‐2017.pdf?ext=.pdf

[16] Mavrides E , Allard S , Chandraharan E *et al*. Prevention and management of postpartum haemorrhage. BJOG An Int J Obstet Gynaecol 2016; 124: e106–e149.

[17] World Health Organization . WHO Recommendations for the Prevention and Treatment of Postpartum Haemorrhage. Geneva: World Health Organization, 2012. 23586122

[18] Oyelese Y , Ananth CV . Postpartum hemorrhage: Epidemiology, risk factors, and causes. Clin Obstet Gynecol 2010; 53(1): 147–156. 20142652 10.1097/GRF.0b013e3181cc406d

[19] Steyerberg EW , Moons KGM , van der Windt DA *et al*. Prognosis research strategy (PROGRESS) 3: Prognostic model research. PLoS Med 2013; 10(2): e1001381. 23393430 10.1371/journal.pmed.1001381 PMC3564751

[20] Brent JC . Making it easy to do it right. N Engl J Med 2001; 345(13): 991–993. 11575294 10.1056/NEJM200109273451311

[21] Kleinrouweler CE , Cheong‐See FM , Collins GS , Kwee A , Thangaratinam S , Khan KS , et al. Prognostic models in obstetrics: Available, but far from applicable. Am J Obstet Gynecol [Internet]. 2016;214(1):79‐90.e36. 10.1016/j.ajog.2015.06.013 26070707

[22] Neary C , Naheed S , McLernon DJ , Black M . Predicting risk of postpartum haemorrhage: A systematic review. BJOG An Int J Obstet Gynaecol 2021; 128(1): 46–53. 10.1111/1471-0528.16379 32575159

[23] Page MJ , McKenzie JE , Bossuyt PM *et al*. The PRISMA 2020 statement: An updated guideline for reporting systematic reviews. BMJ 2021; 372: n71 10.1136/bmj.n71. 33782057 PMC8005924

[24] Licqurish S , Nicolson A , Mol B , Carr B , Foolardi E , Jahanfar S . Risk factors and predictive models for primary postpartum haemorrhage: A systematice review. PROSPERO 2020;(CRD42020136926):1–4. Available from: https://www.crd.york.ac.uk/prospero/display_record.php?ID=CRD42020136926

[25] Moons KGM , de Groot JAH , Bouwmeester W *et al*. Critical appraisal and data extraction for systematic reviews of prediction modelling studies: The CHARMS checklist. PLoS Med 2014; 11(10): e1001744. 25314315 10.1371/journal.pmed.1001744 PMC4196729

[26] Wolff RF , Moons KGM , Riley RD *et al*. PROBAST: A tool to assess the risk of bias and applicability of prediction model studies. Ann Intern Med 2019; 170(1): 51–58. 30596875 10.7326/M18-1376

[27] Meher S , Cuthbert A , Kirkham JJ *et al*. Core outcome sets for prevention and treatment of postpartum haemorrhage: An international Delphi consensus study. BJOG An Int J Obstet Gynaecol 2019; 126(1): 83–93. 10.1111/1471-0528.15335 29920912

[28] Charbit B , Mandelbrot L , Samain E *et al*. The decrease of fibrinogen is an early predictor of the severity of postpartum hemorrhage. J Thromb Haemost 2007; 5(2): 266–273. 17087729 10.1111/j.1538-7836.2007.02297.x

[29] Cortet M , Maucort‐Boulch D , Deneux‐Tharaux C *et al*. Severity of post‐partum hemorrhage after vaginal delivery is not predictable from clinical variables available at the time post‐partum hemorrhage is diagnosed. J Obstet Gynaecol Res 2015; 41(2): 199–206. 25303234 10.1111/jog.12528

[30] Gayat E , Resche‐Rigon M , Morel O *et al*. Predictive factors of advanced interventional procedures in a multicentre severe postpartum haemorrhage study. Intensive Care Med 2011; 37(11): 1816–1825. 21805157 10.1007/s00134-011-2315-0

[31] Wu Q , Yao K , Liu Z *et al*. Radiomics analysis of placenta on T2WI facilitates prediction of postpartum haemorrhage: A multicentre study. EBioMedicine [Internet] 2019; 50: 355–365. 10.1016/j.ebiom.2019.11.010. 31767539 PMC6921361

[32] Koopmans CM , Van Der Tuuk K , Groen H *et al*. Prediction of postpartum hemorrhage in women with gestational hypertension or mild preeclampsia at term. Acta Obstet Gynecol Scand 2014; 93(4): 399–407. 24575790 10.1111/aogs.12352

[33] Rubio‐Álvarez A , Molina‐Alarcón M , Arias‐Arias Á , Hernández‐Martínez A . Development and validation of a predictive model for excessive postpartum blood loss: A retrospective, cohort study. Int J Nurs Stud [Internet] 2018; 79: 114–121. 10.1016/j.ijnurstu.2017.11.009. 29223625

[34] Prata N , Hamza S , Bell S *et al*. Inability to predict postpartum hemorrhage: Insights from Egyptian intervention data. BMC Pregnancy Childbirth 2011; 11(97): 1–10. 22123123 10.1186/1471-2393-11-97 PMC3276439

[35] Peyvandi F , Biguzzi E , Franchi F *et al*. Elevated prepartum fibrinogen levels are not associated with a reduced risk of postpartum hemorrhage. J Thromb Haemost 2012; 10(7): 1451–1453. 22520037 10.1111/j.1538-7836.2012.04755.x

[36] Helman S , Drukker L , Fruchtman H *et al*. Revisit of risk factors for major obstetric hemorrhage: Insights from a large medical center. Arch Gynecol Obstet 2015; 292(4): 819–828. 25903520 10.1007/s00404-015-3725-y

[37] Tsu VD . Antenatal screening: Its use in assessing obstetric risk factors in Zimbabwe. J Epidemiol Community Health 1994; 48(3): 297–305. 8051531 10.1136/jech.48.3.297 PMC1059963

[38] Chen JS , Roberts CL , Simpson JM , Ford JB . Use of hospitalisation history (lookback) to determine prevalence of chronic diseases: Impact on modelling of risk factors for haemorrhage in pregnancy. BMC Med Res Methodol 2011; 11(68): 1–9. 21575257 10.1186/1471-2288-11-68 PMC3120808

[39] Sittiparn W , Siwadune T . Risk score for prediction of postpartum hemorrhages in normal labor at Chonburi hospital. J Med Assoc Thai 2017; 100(4): 382–388. 29911829

[40] Niepraschk‐von Dollen K , Bamberg C , Henkelmann A *et al*. Predelivery maternal fibrinogen as a predictor of blood loss after vaginal delivery. Arch Gynecol Obstet 2016; 294(4): 745–751. 26899183 10.1007/s00404-016-4031-z

[41] Biguzzi E , Franchi F , Ambrogi F *et al*. Risk factors for postpartum hemorrhage in a cohort of 6011 Italian women. Thromb Res [Internet] 2012; 129(4): e1–e7. 10.1016/j.thromres.2011.09.010. 22018996

[42] Dunkerton SE , Jeve YB , Walkinshaw N *et al*. Predicting postpartum hemorrhage (PPH) during cesarean delivery using the Leicester PPH predict tool: A retrospective cohort study. Am J Perinatol 2018; 35(2): 163–169. 28847038 10.1055/s-0037-1606332

[43] Sei K , Masui K , Sasa H , Furuya K . Size of uterine leiomyoma is a predictor for massive haemorrhage during caesarean delivery. Eur J Obstet Gynecol Reprod Biol [internet] 2018; 223: 60–63. 10.1016/j.ejogrb.2018.02.014. 29494995

[44] Suta J , Leungratsameerung S , Phaloprakarn C . A risk score for predicting postpartum hemorrhage in association with cesarean delivery. Thai J Obstet Gynaecol 2015; 23(1): 3–11.

[45] Lee JY , Ahn EH , Kang S *et al*. Scoring model to predict massive post‐partum bleeding in pregnancies with placenta previa: A retrospective cohort study. J Obstet Gynaecol Res 2018; 44(1): 54–60. 29067758 10.1111/jog.13480

[46] Shinohara S , Okuda Y , Hirata S . Association between birth weight and massive haemorrhage in pregnancy with a low‐lying placenta: A 9‐year single‐Centre retrospective cohort study in Japan. J Obstet Gynaecol (Lahore) [Internet] 2019; 39: 22–26. 10.1080/01443615.2018.1454413. 29884097

[47] Moons KGM , Royston P , Vergouwe Y *et al*. Prognosis and prognostic research: What, why, and how? BMJ 2009; 338(7706): 1317–1320.

[48] Moons KGM , Altman DG , Reitsma JB *et al*. Transparent reporting of a multivariable prediction model for individual prognosis or diagnosis (TRIPOD): explanation and elaboration. Ann Intern Med 2015; 162(1): W1–W73. 25560730 10.7326/M14-0698

