# Supplementary material for: Predicting postpartum haemorrhage: A systematic review of prognostic models
Source: Aust N Z J Obstet Gynaecol. 2022 Aug 2;62(6):813–25. doi: 10.1111/ajo.13599 (PMC10087871; doi:10.1111/ajo.13599)
Supplement: Supplementary file 1 — Table S1. Inclusion and exclusion criteria. [file AJO-62-813-s003.docx]

**Table S1: Inclusion and exclusion criteria**

| Inclusion criteria | Exclusion Criteria |
| --- | --- |
| Any quantitative study design  Risk prediction models for primary PPH  Any date range  Published in English  Pregnant women | Qualitative studies, conference presentations, abstracts or dissertations  Risk prediction models for other maternal conditions  Only risk factors for PPH reported  Risk prediction for PPH >24 hours post birth  Not published in English  Non-pregnant women |
