# Supplementary material for: Predicting postpartum haemorrhage: A systematic review of prognostic models
Source: Aust N Z J Obstet Gynaecol. 2022 Aug 2;62(6):813–25. doi: 10.1111/ajo.13599 (PMC10087871; doi:10.1111/ajo.13599)
Supplement: Supplementary file 2 — Table S2. Source of data and characteristic of studies used to develop models for predicting postpartum haemorrhage. [file AJO-62-813-s001.docx]

**Table S2: Source of data and characteristic of studies used to develop models for predicting postpartum haemorrhage**

| Study | Study Setting | Study Dates | Source of data | Type of Prediction Modelling Study |
| --- | --- | --- | --- | --- |
| Chen et al., 2011^37^ | Multi-centre, all births in NSW, Australia | Jul 2005 – Dec 2006 | Retrospective cohort | Development without external validation |
| Helman et al., 2015^35^ | Single-centre, large obstetric centre, Israel | Jul 2005 – Feb 2014 | Retrospective case control | Development without external validation |
| Koopmans et al., 2014^31^ | Multi-centre, six academic and 32 non-academic hospitals in The Netherlands | Oct 2005 – Mar 2008 | Cohort using data from HYPITAT trial | Development without external validation |
| Prata et al., 2011^33^ | Multi-centre, three university hospitals in Egypt | 6 month period in 2004 | Secondary analysis from prospective cohort study | Development without external validation |
| Biguzzi et al., 2012^40^ | Single centre Obstetric unit, Northern Italy | Jul 2007 – Sep 2009 | Prospective cohort | Development without external validation |
| Peyvandi et al., 2012^34^ | Single centre Obstetric unit, Northern Italy | Jul 2007 – Sep 2009 | Secondary analysis from prospective cohort study | Development without external validation |
| Niepraschk-von Dollen et al., 2016^39^ | Single-centre, tertiary centre in Berlin, Germany | Dec 2011 – May 2013 | Prospective cohort | Development without external validation |
| Rubio-Alvarez et al., 2018^32^ | Single-centre, maternity hospital, Spain | Development: 2009 – 2011  Validation: 2013 – 2014 | Retrospective cohort (development) Prospective cohort (validation) | Development with external validation in independent data |
| Tsu, 1994^36^ | Single-centre, hospital in Harare, Zimbabwe | Eight months during 1989 | Retrospective analysis from case control study | Development without external validation |
| Sittiparn & Siwadune, 2017^38^ | Single-centre, maternity hospital, Chonburi, Thailand | Jul 2013 – Jun 2014 | Retrospective cohort | Development without external validation |
| Suta et al., 2015^43^ | Single-centre, maternity hospital in Bangkok, Thailand | Sep 2011 – Dec 2013 | Retrospective cohort | Development without external validation |
| Dunkerton et al., 2017^41^ | Single-centre, tertiary maternity unit, Leicester, UK | Jan 2003 – Dec 2013 | Retrospective cohort | Development without external validation |
| Lee et al., 2018^44^ | Single-centre, maternity unit in South Korea | Jan 2006 – Apr 2016 | Retrospective cohort | Development without external validation |
| Sei et al., 2018^42^ | Single-centre, maternity hospital, Saitama, Japan | Jan 2005 – Dec 2011 | Retrospective cohort | Development without external validation |
| Shinohara et al., 2018^45^ | Single-centre, maternity hospital, Yamanashi, Japan | Jan 2008 – Dec 2016 | Retrospective cohort | Development without external validation |
| Wu et al., 2019^30^ | Multi-centre, two maternity hospitals in Zhengzhou, China | May 2013 – Apr 2019 | Retrospective cohort | Development with external validation in independent data |
