# Supplementary material for: Predicting postpartum haemorrhage: A systematic review of prognostic models
Source: Aust N Z J Obstet Gynaecol. 2022 Aug 2;62(6):813–25. doi: 10.1111/ajo.13599 (PMC10087871; doi:10.1111/ajo.13599)
Supplement: Supplementary file 3 — Table S3. Search terms. [file AJO-62-813-s002.docx]

**Table S3: Search terms**

**Keywords from papers found from initial searching**

| **Postpartum Haemorrhage** | **Vaginal birth** | **Risk factors** |
| --- | --- | --- |
| Postpartum hemorrhage  Post-partum haemorrhage  Post-partum hemorrhage  PPH  Primary postpartum haemorrhage  Primary postpartum haemorrhage  Uterine Atony  Blood loss  Postpartum Blood loss  Severe adverse maternal morbidity  Haemorrhagic shock  Obstetric haemorrhage  Obstetric haemorrhage  Maternal morbidity  Peripartum haemorrhage  Peripartum haemorrhage  Haemorrhage  Hemorrhage  Severe postpartum haemorrhage  Severe postpartum hemorrhage | Vaginal delivery  Third stage of Labour  Third stage of Labor  Normal labor  Normal labour  Perinatal outcome  Normal delivery | Predictive model  Validation  Risk Score  Prediction  Prognostic model  Predictive value  Receiver operating characteristic curve  Predictive score |

**MeSH on Demand suggestions:**

[Pregnancy](https://meshb.nlm.nih.gov/record/ui?name=Pregnancy)

[Female](https://meshb.nlm.nih.gov/record/ui?name=Female)

[Humans](https://meshb.nlm.nih.gov/record/ui?name=Humans)

[Postpartum Hemorrhage](https://meshb.nlm.nih.gov/record/ui?name=Postpartum%20Hemorrhage)

[Placenta, Retained](https://meshb.nlm.nih.gov/record/ui?name=Placenta,%20Retained)

[Risk Factors](https://meshb.nlm.nih.gov/record/ui?name=Risk%20Factors)

[Episiotomy](https://meshb.nlm.nih.gov/record/ui?name=Episiotomy)

[Prevalence](https://meshb.nlm.nih.gov/record/ui?name=Prevalence)

[Parity](https://meshb.nlm.nih.gov/record/ui?name=Parity)

[Nomograms](https://meshb.nlm.nih.gov/record/ui?name=Nomograms)

[Postpartum Period](https://meshb.nlm.nih.gov/record/ui?name=Postpartum%20Period)

[Delivery, Obstetric](https://meshb.nlm.nih.gov/record/ui?name=Delivery,%20Obstetric)

[Hemoglobins](https://meshb.nlm.nih.gov/record/ui?name=Hemoglobins)

[Body Weight](https://meshb.nlm.nih.gov/record/ui?name=Body%20Weight)

[Adult](https://meshb.nlm.nih.gov/record/ui?name=Adult)

[Parity](https://meshb.nlm.nih.gov/record/ui?name=Parity)

[Oxytocin](https://meshb.nlm.nih.gov/record/ui?name=Oxytocin)

[Postpartum Hemorrhage](https://meshb.nlm.nih.gov/record/ui?name=Postpartum%20Hemorrhage)

[Retrospective Studies](https://meshb.nlm.nih.gov/record/ui?name=Retrospective%20Studies)

[Incidence](https://meshb.nlm.nih.gov/record/ui?name=Incidence)

[Multivariate Analysis](https://meshb.nlm.nih.gov/record/ui?name=Multivariate%20Analysis)

[Reproductive History](https://meshb.nlm.nih.gov/record/ui?name=Reproductive%20History)

[Mothers](https://meshb.nlm.nih.gov/record/ui?name=Mothers)

[Linear Models](https://meshb.nlm.nih.gov/record/ui?name=Linear%20Models)

[Parturition](https://meshb.nlm.nih.gov/record/ui?name=Parturition)

[Labor, Obstetric](https://meshb.nlm.nih.gov/record/ui?name=Labor,%20Obstetric)

[Maternal Age](https://meshb.nlm.nih.gov/record/ui?name=Maternal%20Age)

[Labor, Induced](https://meshb.nlm.nih.gov/record/ui?name=Labor,%20Induced)

[Postpartum Period](https://meshb.nlm.nih.gov/record/ui?name=Postpartum%20Period)

**Updated review search terms used:**

algorithm

area under the curve

auc

c statistic

c-statistic

caesarean section

calibration

cesarean

discriminat*

forceps

delivery

hemorrhage

incidence

indices

linear

models

multivariable

multivariate

analysis

nomogram*

normal birth

labor

labour

perinatal

outcome

prediction

predictive

model

value

prevalence

prognostic

score

retrospective

study

risk factors

roc

stratification

third stage

vacuum

vaginal birth

ventouse
